# From Buildings to the Environment: Flows, Exposure Pathways, and Toxicological Relevance of Tris(2-Chloro-1-Methylethyl) Phosphate (TCPP), Diuron, and Bis(2-(Perfluorohexyl)Ethyl) Phosphate (6:2 diPAP)

**DOI:** 10.3390/toxics14090811

**Published:** 2026-09-11

**Authors:** Jolita Kruopienė, Edgaras Stunžėnas, Jenny Fäldt, Riikka K. Vainio

**Affiliations:** 1 Institute of Environmental Engineering, Kaunas University of Technology, 44239 Kaunas, Lithuania; edgaras.stunzenas@ktu.lt; 2 Environment and Health Department, Division for Urban Environment, City of Stockholm, Box 8136, 104 20 Stockholm, Sweden; jenny.faldt@stockholm.se; 3 School of Sustainable Environment, Faculty of Engineering, Turku University of Applied Sciences, 20520 Turku, Finland; riikka.vainio@turkuamk.fi

**Keywords:** substances of concern, buildings, construction, substance flow analysis, organophosphate esters, TCPP, biocides, diuron, per- and polyfluoroalkyl substances (PFAS), 6:2 diPAP

## Abstract

The use of hazardous chemicals in construction materials raises concerns regarding their release into the environment and their potential impact on ecosystems and human health. This study investigated the flows of tris(2-chloro-1-methylethyl) phosphate (TCPP), diuron, and bis(2-(perfluorohexyl)ethyl) phosphate (6:2 diPAP) from buildings to environmental compartments and evaluated their toxicological relevance through environmental occurrence and exposure pathways. A quantitative substance flow analysis was performed for TCPP, while conceptual flow models were developed for diuron and 6:2 diPAP. TCPP flow analysis showed that approximately 70,804 t/year accumulated in products, primarily rigid polyurethane insulation materials, alongside continuous environmental releases into indoor dust, soils, and aquatic systems. It resulted in annual accumulation of nearly 444 t of TCPP in soils and 64 t in aquatic systems. Diuron was identified as a source of aquatic exposure through rainfall-driven wash-off, whereas 6:2 diPAP exhibited indoor and outdoor emission pathways and transformation into persistent perfluoroalkyl carboxylic acids. Field measurements of Interreg NonHazCity3 project confirmed the occurrence of organophosphate esters, biocides, and PFAS in indoor dust, stormwater, and wastewater. The results demonstrate that buildings act as continuous sources of toxicologically relevant substances, pointing to the necessity of upstream source control, the prevention of regrettable chemical substitution, and enhanced transparency in construction materials to mitigate long-term environmental and health risks.

## 1. Introduction

The use of hazardous chemicals in the construction sector raises concerns about their potential negative impact on the environment and human health [1,2,3]. Some of these substances, although they perform important technical functions, are persistent in the environment, toxic, or tend to accumulate in organisms, and are therefore substances of concern. Efforts are being made to reduce the use of such compounds. Buildings deserve special attention due to their long lifecycle, large-scale use of construction materials and direct contact with the environment and humans.

Various stakeholders are active in addressing these issues. Research initiatives aim to identify substances of concern and understand their pathways from buildings to the environment [3,4,5,6,7,8]. Industry, together with researchers, is working toward safer alternatives [9,10]. Policymakers develop legislation and strategies, such as the EU Construction Products Regulation (CPR), REACH, and Biocidal Products Regulation (BPR), intended to ensure transparency and safety throughout the product lifecycle [11,12,13]. Complementary instruments and practices, such as building certification schemes (e.g., LEED and BREEAM internationally, DGNB and Miljöbyggnad mainly in the Baltic Sea region [14,15,16,17]), environmental product declarations (EPDs), eco-labels, green public procurement, and digital databases (e.g., Byggvarubedomningen—BVB, Basta [18,19]), are used to support decision-making and encourage the transition toward non-hazardous construction.

A screening study conducted under the Interreg project NonHazCity3 [20,21] examined stormwater, indoor dust and air, residential wastewater, and construction products in five Baltic Sea region cities (Helsinki, Stockholm, Västerås, Tallinn, and Turku) [22]. The findings revealed contamination by organic pollutants, including plasticizers, per- and polyfluoroalkyl substances (PFAS), chlorinated paraffins, and organophosphate esters (OPEs), with some compounds reaching up to 0.1% by weight in indoor dust. Stormwater was shown to act as an important pathway for pollutants between built and natural environments, containing biocides, OPEs, metals, and PFAS, with notable variations across cities and building types. Biocides such as diuron, propiconazole, and mecoprop were detected in runoff from areas with new wooden claddings, highlighting the environmental impact of facade treatments. PFAS were present in multiple matrices, with significant differences in concentrations among locations. The study also noted a trend toward replacing older hazardous substances with emerging chemicals of concern [22].

Based on these results, three representative compounds were selected for detailed analysis: the organophosphate ester tris(2-chloro-1-methylethyl) phosphate (TCPP), the biocide diuron, and the PFAS compound bis(2-(perfluorohexyl)ethyl) phosphate (6:2 diPAP).

### 1.1. Tris(2-Chloro-1-Methylethyl) Phosphate (TCPP)

The nomenclature for the OPE identified by CAS 13674-84-5 is characterized by significant variability in the scientific literature and regulatory frameworks, reflecting its complex isomeric composition. The substance is most frequently referred to as tris(2-chloro-1-methylethyl) phosphate or tris(1-chloro-2-propyl) phosphate, with abbreviations varying mostly between TCPP and TCIPP (TCPP will be used further in the article). The European Commission’s Risk Assessment Report [23] and the European Chemical Agency (ECHA) Screening Report [24] primarily utilized the designation tris(2-chloro-1-methylethyl) phosphate (TCPP) (CAS 13674-84-5; EC 237-158-7). The US National Toxicology Program (NTP) Technical Report 602 [25] adopted the name tris(chloropropyl) phosphate (TCPP) to encompass the isomeric mixture. The NTP identifies the most abundant constituent (50–85%) as tris(1-chloro-2-propyl) phosphate (CAS 13674-84-5) and abbreviates it as TCIPP. Subsequently, regulatory management under REACH (e.g., registration dossiers) has transitioned toward more comprehensive identifiers, such as EC 807-935-0, which describes the substance as a “reaction product” of several structural isomers. This reflects the fact that the commercial substance is a complex mixture rather than a pure compound.

TCPP is used as an additive flame retardant and plasticizer due to its low volatility, thermal stability, and polymer compatibility [23]. The predominant usage is in spray polyurethane (PUR) foams and rigid foams for building insulation, as well as in flexible foams for furniture or the automotive industry [23,24].

Because TCPP is physically added rather than chemically bound to polymer matrices, it gradually leaches, abrades, or volatilizes during the product lifecycle, resulting in its detection in indoor air, dust, wastewater, surface waters, sediments, and human matrices [4,26,27,28,29,30,31,32].

Initially adopted as a safer alternative to restricted brominated flame retardants and tris(2-chloroethyl) phosphate (TCEP), TCPP gradually attracted increasing regulatory attention as new toxicological data became available. In 2018, ECHA published a screening report evaluating whether TCPP in articles, particularly upholstered furniture and childcare products, should be restricted [24]. Additional concerns arose from the US National Toxicology Program (NTP) Technical Report 602, which evaluated an isomeric mixture of chloropropyl phosphate esters and reported evidence of carcinogenic activity in experimental animals [25]. On this basis, ECHA identified TCPP as a candidate for further consideration under the REACH restriction process, although no finalized restriction targeting TCPP has yet been adopted.

### 1.2. Diuron

Diuron (3-(3,4-dichlorophenyl)-1,1-dimethylurea; CAS 330-54-1; EC 206-354-4) was originally developed as an agricultural herbicide. Subsequently, it was introduced into construction materials as a biocidal active substance to prevent the growth of algae on building surfaces.

Because diuron is physically mixed into coating formulations rather than chemically bound, it is gradually released during rainfall events. This rainfall-driven wash-off enters urban stormwater systems and wastewater treatment plants, ultimately reaching surface waters, sediments, and occasionally groundwater [3,5,6,8,33,34,35,36,37,38,39].

Environmental concern is primarily associated with the high toxicity of diuron towards algae and aquatic plants [31]. Even though it is persistent, diuron is not completely inert, and undergoes biotic and abiotic transformation, where metabolites 1-(3,4-dichlorophenyl)-3-methylurea (DCPMU), 1-(3,4-dichlorophenyl)urea (DCPU), and 3,4-dichloroaniline (3,4-DCA) are generated [32,33]. 3,4-DCA is of particular environmental concern because it exhibits greater ecotoxicity than the parent compound and may persist in soils and aquatic environments.

Due to environmental risks, diuron’s approval for use in plant protection products was not renewed in the European Union [34]. However, it continues to be evaluated under the Biocidal Products Regulation for film (PT7) and construction material (PT10) preservation [13]. Diuron is listed as a priority substance for water quality in the European Union [35].

### 1.3. Bis(2-(Perfluorohexyl)Ethyl) Phosphate (6:2 diPAP)

Bis(2-(perfluorohexyl)ethyl) phosphate (CAS 57677-95-9; EC 975-653-6), commonly abbreviated as 6:2 diPAP, belongs to the class of fluorotelomer phosphorus esters (PAPs), which are a subset of the PFAS family. PAPs are considered PFAS precursors because they can undergo abiotic and biotic transformation into highly persistent perfluoroalkyl carboxylic acids (PFCAs) [36,37].

The exceptional strength of the carbon–fluorine bonds provides these compounds with high thermal and chemical stability, as well as resistance to water, oils, stains, and dirt. It is added to indoor and outdoor paints, floor coverings, carpets, and specialized floor waxes or polishes [7,38,39].

Used as an additive, 6:2 diPAP migrates from treated products during their service life [38]. Indoors, volatilization and abrasion drive its presence in dust and indoor air [29,40,41,42,43]. 6:2 diPAP has also been quantified across various outdoor matrices: municipal landfill leachates [44], municipal sewage sludge [45], and aquatic sediments [46]. 6:2 diPAP detection in wildlife tissues (worms) and human blood matrices confirm widespread environmental distribution and systemic bioaccumulation [46].

Regulatory attention toward 6:2 diPAP has increased with growing concern regarding PFAS as a chemical class. 6:2 diPAP is covered by the universal PFAS restriction proposal submitted to ECHA in 2023, reflecting concerns regarding persistence, transformation into terminal PFAS, and long-term environmental exposure [47].

### 1.4. Aim of Research

These three substances illustrate different functional roles in construction materials and different environmental behaviors; all three have been detected in indoor and/or outdoor environments. Their toxicological relevance differs across substances: TCPP displays significant chronic toxicity and carcinogenic potential, diuron is of particular concern because of its high acute toxicity to primary aquatic producers, while 6:2 diPAP is relevant as a precursor that can contribute to the formation and accumulation of highly persistent perfluoroalkyl carboxylic acids. As such, the three substances provide complementary case studies for linking chemical flows from buildings with environmental exposure and toxicological significance.

The aim of the study was to analyze the flows and release pathways associated with the use of TCPP, diuron, and 6:2 diPAP in construction products and to evaluate their toxicological relevance alongside environmental occurrence. By combining substance flow analysis and conceptual pathway modeling with previously reported environmental screening data, the study relates estimated and qualitatively identified emission pathways to observed substance occurrence in indoor and outdoor environments, and discusses their implications for human and environmental exposure. Rather than offering a full quantitative risk assessment, the study provides a screening-level assessment of the environmental and health significance of building-related chemical emissions.

## 2. Materials and Methods

The study integrates a few types of evidence: a quantitative substance flow analysis (SFA) for TCPP, conceptual flow models for diuron and 6:2 diPAP, environmental measurements generated within the NonHazCity3 project, and targeted literature evidence on the occurrence, release, environmental fate, and exposure pathways of the selected substances.

The NonHazCity3 measurements were used as empirical evidence, a “reality check” in the discussion, to assess whether the pathways identified through the flow analysis and conceptual models are consistent with observed substance occurrence. Because these measurements were previously reported, the full analytical methodology is available in [22] and is not duplicated here.

In addition to quantifying or conceptually describing substance flows, the identified pathways were interpreted from an exposure and toxicological perspective. Environmental occurrence data from the literature and the NonHazCity3 screening campaign were compared with available information on toxicity, persistence, transformation and relevant environmental quality criteria. This comparison was used to identify pathways of particular toxicological relevance and potential exposure scenarios. The study was not designed to derive human health or environmental risk estimates: the assessment is considered a screening-level evaluation.

### 2.1. TCPP Flow Analysis

The analysis was conducted using the STAN 2.6 software (TU Wien, Vienna, Austria) [48,49], following the mass balance principle. The geographical scope is Europe, with a temporal scale encompassing one year.

Pre-existing stocks within the system were excluded due to their high degree of uncertainty; thus, the analysis focuses exclusively on annual TCPP inputs, transient flows, and the resulting stock increments within the defined timeframe. Input data for TCPP production and market supply were sourced from recent regulatory reports, and cover July 2022 to June 2023. Sectoral distributions and release rates were derived from the EU Risk Assessment Report [23]. Transfer between environmental compartments was defined based on distribution according to fugacity modeling, as presented in [23] and similarly in [50,51], and supported by established literature on TCPP’s environmental fate and partitioning behavior between environmental compartments [26,27,28,52,53].

To estimate uncertainty in TCPP flows, the uncertainty concept, which categorizes data sources, was used as described and utilized in the previous publications [54,55,56]. Data sources were categorized into three groups: input data from the recent regulatory documents [57,58] were assigned to uncertainty level 1 (low uncertainty) and received a value of 15%. Data on TCPP distribution among various use categories, as well as release rates, were assigned to uncertainty level 2 (average) and received a value of 30%. Substance distribution among environmental compartments was attributed to the higher uncertainty level 3 and received a value of 60%. Although data were entered with uncertainties and data reconciliation was performed, uncertainties are not indicated in the presented diagrams for the sake of visual clarity.

Table S1 in the Supplementary Materials details all TCPP flows, their initial and reconciled vales, underlying assumptions, data sources, and uncertainties.

All numbers in figures, representing TCPP flows and accumulations, are presented to two decimal places. This is a compromise between the need to show the magnitude of flows, the change in flows, and the technical capabilities of STAN software. The software allows for either a fixed number of decimal places or a format based on significant figures. The latter results in greater rounding of the largest values, which can obscure small changes in the mass balance because the smaller flows have only a minor effect on the larger flows. Unfortunately, when displaying two decimal places, the numerical value of some flows appears to be zero, although the flows are not in fact zero. The full numbers can be found in Table S1 of the Supplementary Material.

### 2.2. Diuron and 6:2 diPAP Flow Analysis

Conceptual material flow models were established for diuron and 6:2 diPAP. Because of the lack of detailed data on the amounts of these substances produced, imported, or distributed across specific product formulations, the large variability in emission factors and transfer coefficients for diuron, and a lack of data for 6:2 diPAP, a full quantitative mass balance was unfeasible.

Instead, the scientific literature was used to identify and substantiate relevant release, flows, and exposure pathways for diuron [3,5,8,22,31,43,59,59,60,61,62,63,64,65,66,67,68,69,70,71,72,73] and for 6:2 diPAP [22,29,36,37,39,41,42,44,45,46,74,75,76,77,78,79,80,81,82,83,84,85,86]. Literature evidence was identified through targeted searches and screening of relevant publications and reference lists. The aim was not to conduct a systematic or comprehensive review of the available data. Publications were selected based on their ability to inform specific release, transport or exposure pathways. European studies were prioritized where available, while non-European studies were retained where they provided relevant evidence for pathways or compartments for which European data were limited.

## 3. Results

This chapter presents the results of the TCPP flow analysis alongside the conceptual flow models for diuron and 6:2 diPAP. It identifies the dominant release pathways, transfer between environmental compartments, and accumulation in technosphere and the environment. These results will be further discussed in relation to measured environmental occurrence, potential exposure pathways, and the toxicological characteristics of the three substances, thereby providing a basis for evaluating their environmental and human-health relevance.

### 3.1. Results of TCPP Flow Analysis

**Figure 1:**
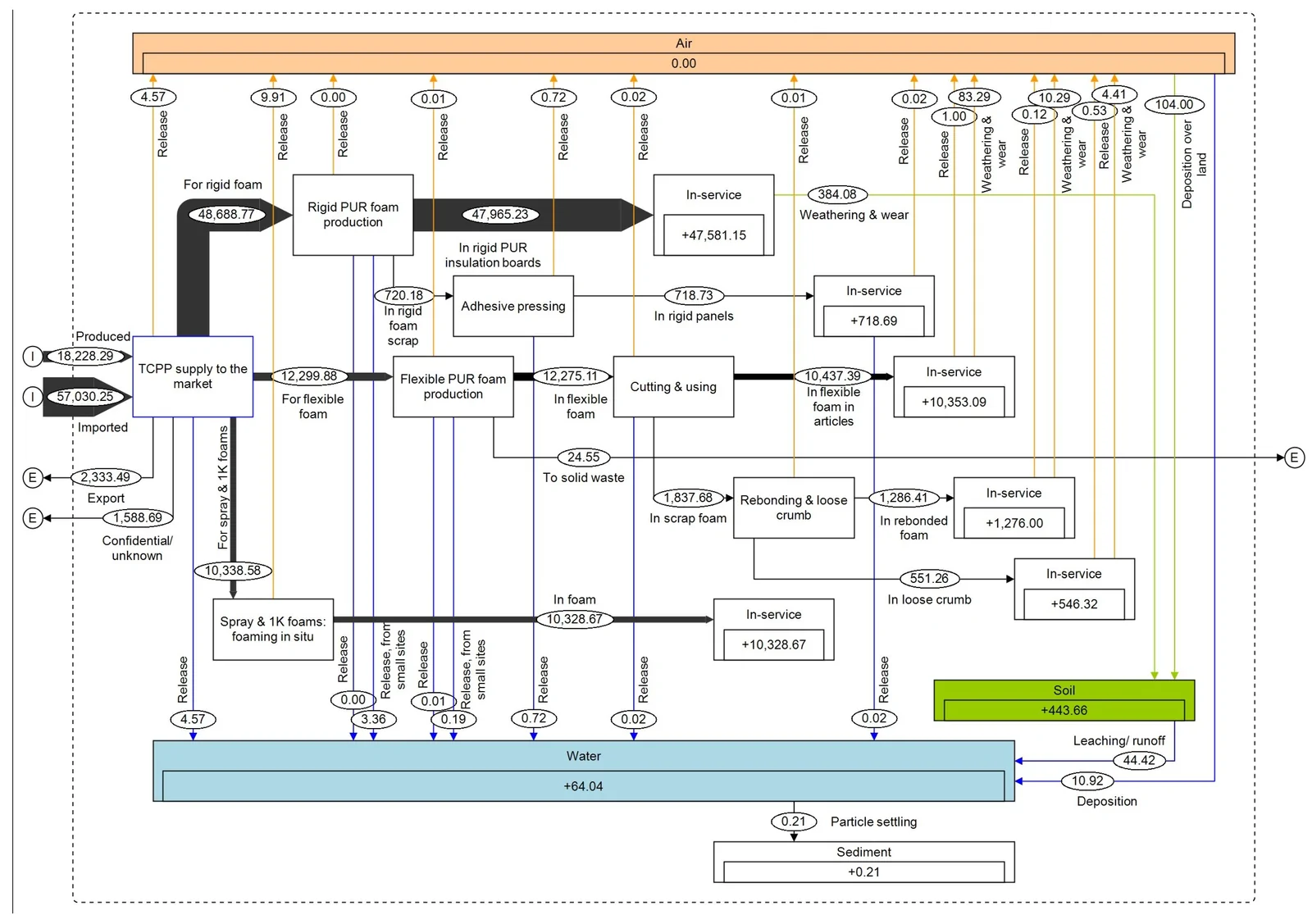
European TCPP flows resulting from one-year inputs. Values are expressed as t TCPP/year. I—import, E—export. More detailed information about all the flows, including uncertainties, is provided in Table S1 of the Supplementary Material.

Figure 1 presents the calculated European TCPP balance resulting from one year of inputs. It is dominated by technospheric accumulation, while environmental releases represent comparatively small but continuous flows.

#### 3.1.1. Flows Related to TCPP Supply to the Market

**Figure 2:**
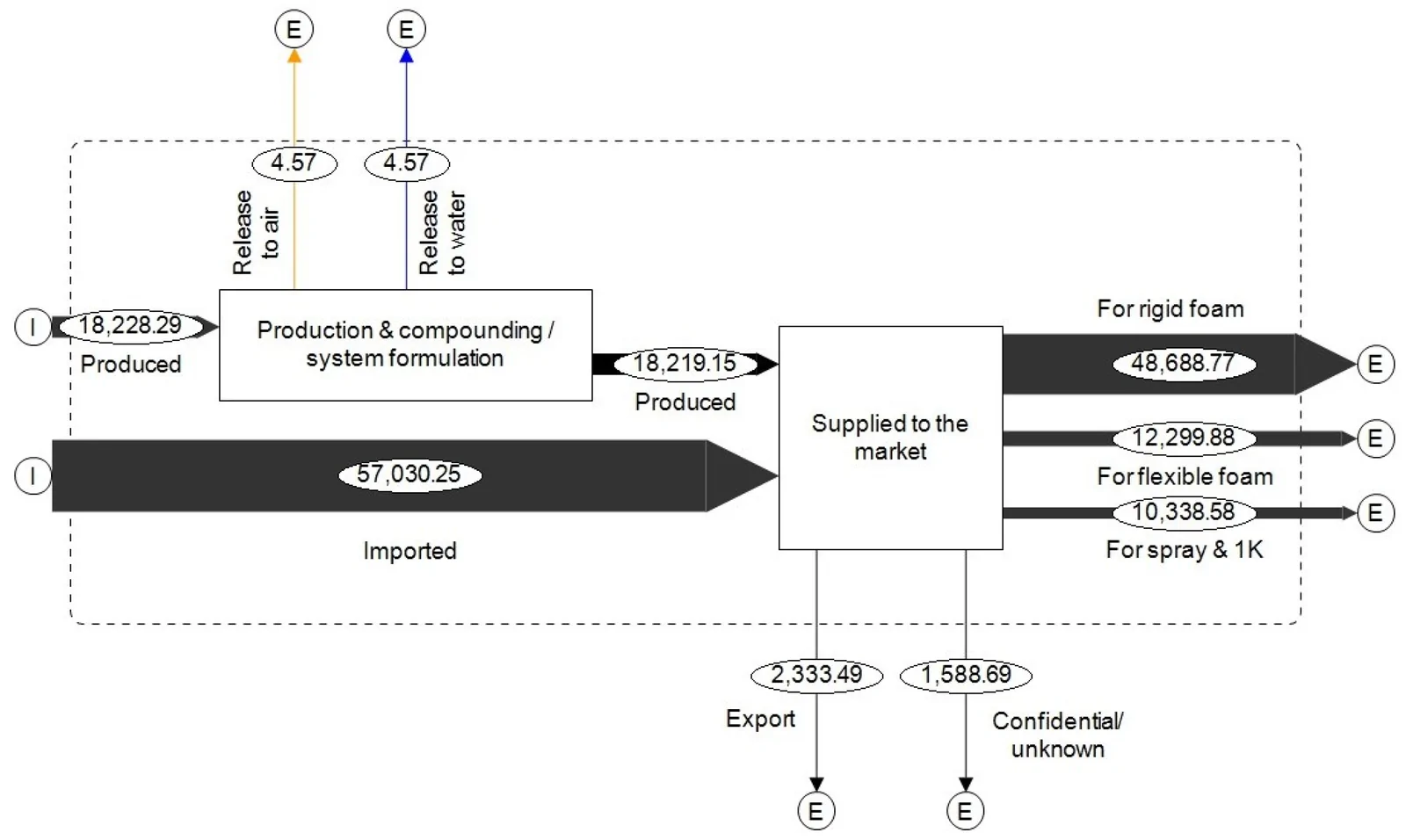
Flows related to TCPP supply to the market. Values are expressed as t TCPP/year. I—import, E—export.

In recent years (see Figure 2), imports accounted for the majority of TCPP input to the European system, accounting for approximately 80% of total supply, mainly originating from the People’s Republic of China [57,58]. Domestic production contributed the remaining share, around 20%, of which approximately 13% was exported. A further 1589 t appear as exports from the system: according to the Risk Assessment Report [23], information on the use of up to 2.5% TCPP was confidential or unknown; this fraction could not be allocated to defined use sectors and is therefore shown as leaving the system boundary.

Releases of TCPP occur at multiple stages of its life cycle, including production, formulation, application, use, and end-of-life management. Emissions during manufacture and PUR system formulation are quantified as 4.57 t/year each to air and water and represent only a very small fraction of the total system throughput, reflecting largely closed and controlled industrial processes.

TCPP for rigid foam applications represent around two thirds, or 48,689 t/year, of the TCPP consumption [23,57]. These foams are primarily used for thermal insulation in the construction industry, as insulation boards, block foams, and laminated sandwich panels.

Spray foams are a subset of rigid foams that are applied on site by professional contractors using high-pressure spray equipment, and form seamless insulation of large surfaces, such as the underside of roofs or the interior of industrial warehouses. One-component foams (1K foams) are usually applied from pressurized aerosol cans intended for both professional and “do-it-yourself” use. 1K foams are suitable for filling gaps, joints, and cracks around window and door frames, as well as for sealing pipe penetrations. Despite some differences, such as professional or individual use, the processes and flows for spray and 1K foams were depicted together, as they both involve similar application methods (spraying) and use stages, and the same release rates. Spray foam and 1K foam consumed the least TCPP (10,339 t/year).

The rest of TCPP, 12,300 t/year, was used for flexible PUR foams. This foam is not used is the construction sector, but mainly in furniture and automotive interiors. Nevertheless, this use of TCPP is also included in the flow analysis to obtain a more complete picture of the situation. Furthermore, furniture use is related to buildings, while the use in the automotive sector could not be separated due to the lack of individual data.

#### 3.1.2. Releases from TCPP Use in Production and Recycling Processes

Emissions to the environment from the lifecycle stages related to processing and recycling amounted to 14.97 t of TCPP per year, of which 10.67 t were released into the air, and the remaining 4.30 t were released into waters.

In the production of both rigid and flexible PUR foams, some release of TCPP occurs during curing and storage both to the air and water. For water, according to [23], the bigger share of releases comes from raw material handling by smaller producers. Scraps and production waste from standard rigid PUR boards, instead of being discarded, undergo an adhesive pressing recycling process where they are crushed into granules, mixed with polyurethane adhesives, and compressed at high temperatures. During this industrial adhesive pressing stage, elevated temperatures and open handling trigger volatile and particulate losses to the air and to wastewater. For flexible PUR foam production, the primary TCPP loss, 24.55 t, arises as solid waste. The produced flexible foam blocks are cut and used for furniture production, which generates dust. Even though dusts are collected at the point of cutting, a small proportion might still escape and hence some TCPP might reach the environment, both air and water, e.g., via cleaning [23]. The remaining flexible scrap foam is collected and partitioned into mechanical recycling pathways, either through a rebonding process or a downcycled loose crumb process. The granulation required for both rebonded and loose crumb foam takes place in contained equipment but may still release some TCPP to the air via dust leakage, while emissions to water are entirely avoided. In the case of foaming in situ, the application phase (spraying) results in TCPP emissions to the air due to the evaporation from the uncovered expanding foam surface prior to curing. 

#### 3.1.3. Releases of TCPP During the In-Service Stage

Once incorporated into the (construction) products, the vast majority of the annual TCPP input accumulates as an anthropogenic stock within the technosphere (70,804 t) while some TCPP escapes as releases into the environment. The release dynamics during this stage are governed by the expected lifetime of the product and whether or not TCPP is enclosed during the in-service stage. Also, as noted in [23], the release of TCPP is limited by the available fraction: for TCPP, 40% of the substance present is available for release.

Some 99.7 t of TCPP was released to the air; 1.68 t of this was due to volatilization from the articles themselves, and 98.0 t was due to weathering and wear. A total of 0.024 t of release went to water, and 384 t went to soil.

TCPP volatilization is expected from the flexible foam, even though the foam is usually covered by fabrics and upholstery. The bigger release factor was used in the case of loose crumb, as it is usually used in outdoor applications, such as garden furniture cushions. Washing and contact of foam with water are unlikely; therefore, there are no releases to water indicated. Consumer products subjected to physical erosion over their service life trigger an additional release pathway: over the service life of flexible, rebonded, and loose crumb foams, physical weathering and wear break away micro-particles of the polymer. These small particles possess a high specific surface area providing good conditions for the locked-up TCPP to volatilize instantaneously upon particle detachment, transferring this entire erosion stream directly into the air.

In the case of rigid foam used as insulation boards, no release is expected, because the boards are effectively sealed within building walls (wall sandwich). However, structural PUR panels pressed from scrap/waste material have a different use profile, such as furniture, sailing boats, and flooring material. Because they lack airtight structural sealing compared to sandwich panels, they exhibit minor continuous volatilization to air and wastewater. Even though direct volatile in-service emissions are virtually non-existent for rigid PUR insulation boards, a rather substantial release to soil can be expected when modifications to the physical form of boards, by cutting them to the required size, need to be made on construction sites.

Air circulation around the fully cured spray foam and 1K foam, covered by protective plaster or frame coverings, is negligible, thus no release is expected.

The expected lifetime of products varies, from 10 years for products with flexible, rebounded, and loose crumb foams, to 25 years for spray foams and 1K foams, 30 years for rigid panels from adhesive pressing, and even 50 years for rigid insulation boards. The model does not include TCPP flows to end-of-life treatment, because no previously accumulated stock was included in the model, while the current stock supplement will leave the system, maybe for landfilling or incineration, only when the lifetime of products is over.

#### 3.1.4. Flows Within Environmental Compartments

A total of 115 t of TCPP was emitted into the air from the yearly input of the substance in Europe, predominantly from the weathering and wear of flexible PUR foams. Once in the atmosphere, both gaseous and particle-bound TCPP is rapidly washed out or deposited. Thus, the atmosphere functions as a transport corridor rather than an accumulation sink.

The majority of the atmospheric load initially deposits onto soil and urban impervious surfaces, while a smaller fraction directly enters surface waters. Once in the terrestrial environment, TCPP is partially transferred into the aquatic environment via surface runoff and wash-off processes. As a result, nearly 444 t/year stays in the soil, while the aquatic compartment accumulates +64 t/year. Within surface waters, a small fraction undergoes particle settling, transitioning into the benthic sediment compartment. Due to the substance’s resistance to rapid abiotic and biotic degradation [23], only a minor fraction of the total input is broken down within a year, resulting in the continuous accumulation of residual TCPP in terrestrial and aquatic compartments [26].

### 3.2. Results of Diuron Flow Analysis

**Figure 3:**
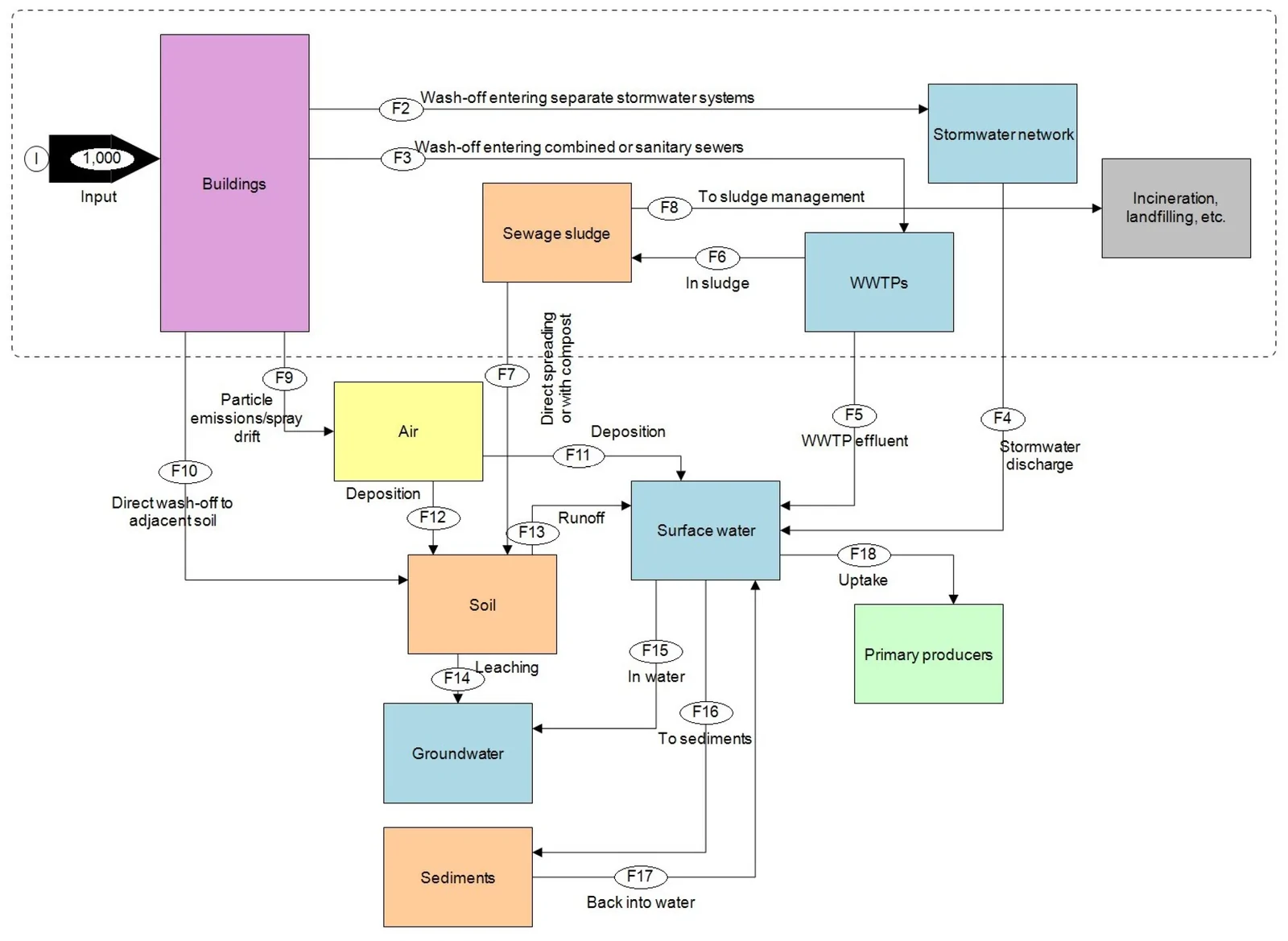
Conceptual flow model for diuron released from buildings. I—input; F—diuron flows. Transformation and degradation processes are not shown. Technosphere is within the dashed box, environment—outside.

A conceptual substance flow model that illustrates the transport pathways of diuron from buildings through the technosphere and the environment is presented in Figure 3. Transformation and degradation processes occurring within the environmental compartments and technosphere are not shown in the model.

The annual consumption of diuron (F1) in the construction sector is estimated at approximately 1000 tonnes. This estimate is in alignment with the REACH registration database, where diuron falls within the 100–1000 t per year tonnage band [31]. Under REACH, the registration of substances in imported articles is only mandatory if the substance is intended to be released under normal or reasonably foreseeable conditions of use [12]. Because the leaching of biocides from building materials is not an intended release, imported diuron-containing articles are not covered by REACH registration and do not fall into the mentioned tonnage band. Even though there are also other market uncertainties, such as potential export or minor alternative applications, construction materials, such as paints, plasters or renders, can be considered the dominant usage of diuron.

Due to its moderate water solubility (42 mg/L), persistence, and low volatility, diuron is gradually washed out from building materials during their service life and enters urban drainage systems. In model-house experiments, concentrations in runoff from freshly treated facade render reached up to 39.5 mg/L during initial runoff events and remained at 1–5 mg/L during subsequent measurements within the first year [5]. Mean concentrations of 900 µg/L and 1000 µg/L were measured in residential facade runoff in Berlin [3] (Table 1). Wash-off enters either separate stormwater systems (F2) or sanitary sewers (F3), eventually reaching surface waters (F4–F5), while direct wash-off to adjacent soils (F10) also occurs.

Because of its low Henry’s constant and vapor pressure, atmospheric transport is negligible and particle emissions and spray drift during application (F9) represent only minor pathways [31]. Nevertheless, atmospheric deposition may contribute to contamination of nearby soils and surface waters (F11–F12). Diuron concentrations of up to 12 µg/L have been reported in urban stormwater, whereas concentrations in municipal WWTP influents and effluents range within 23–68 ng/L and 25–182 ng/L, respectively [8,43,59]. Although wastewater treatment may reduce concentration to some extent, complete removal is generally not achieved. During wastewater treatment, a part of the diuron is transferred to sewage sludge (F6), which may subsequently be applied to agricultural land directly or after composting (F7), whereas the remaining sludge is directed to incineration or landfilling (F8).

According to [60], diuron is detected in soils, sediments, surface waters, and groundwater. In soils, first-order degradation coefficients range from 0.0004 to 0.02 day^−1^, corresponding to half-lives of 35–1733 days [61] while average mineralization reaches 22.2% [62]. Field lysimeter experiments showed that approximately 50% of the applied diuron remained in the upper soil layer after 245 days, whereas less than 0.5% was recovered in leachate water [63]. Additional studies detected only 0–0.27% of the applied diuron in outdoor soil-column leachates, indicating limited but persistent transport to groundwater (F14) [64]. Nevertheless, groundwater contamination is well documented, with concentrations reaching up to 140 ng/L [65].

Soil runoff and erosion (F13) further contribute to contamination of rivers and lakes. Surface water concentrations may reach 8.6 µg/L [8]. Under natural sunlight, diuron degradation half-lives vary between 9 and 38 days, whereas biodegradation in dark river water is considerably slower, with half-lives of 602–814 days [66].

Sediments act as an important environmental sink (F16), containing 0.026–1.7 µg/g in contaminated catchments [67]. Moreover, no measurable degradation was observed in marine sediment microcosms over three months under either aerobic or anaerobic conditions [68], indicating prolonged residence times and possible remobilization back into the water column (F17).

Although diuron has a low bioaccumulation potential (log P_OW_ ≈ 2.89, BCF = 57.1 in the aquatic food chain) [31], its ecological significance is driven primarily by its effects on primary producers (F18). Diuron inhibits photosystem II and can impair photosynthetic activity in algae and aquatic macrophytes [60]. Consequently, the environmental significance of building-related diuron emissions is associated with repeated or episodic exposure of sensitive aquatic organisms in receiving waters. Even where substantial bioaccumulation is not expected, repeated pulses from treated building surfaces may create ecologically relevant exposure conditions in urban receiving waters.

### 3.3. Results of 6:2 diPAP Flow Analysis

**Figure 4:**
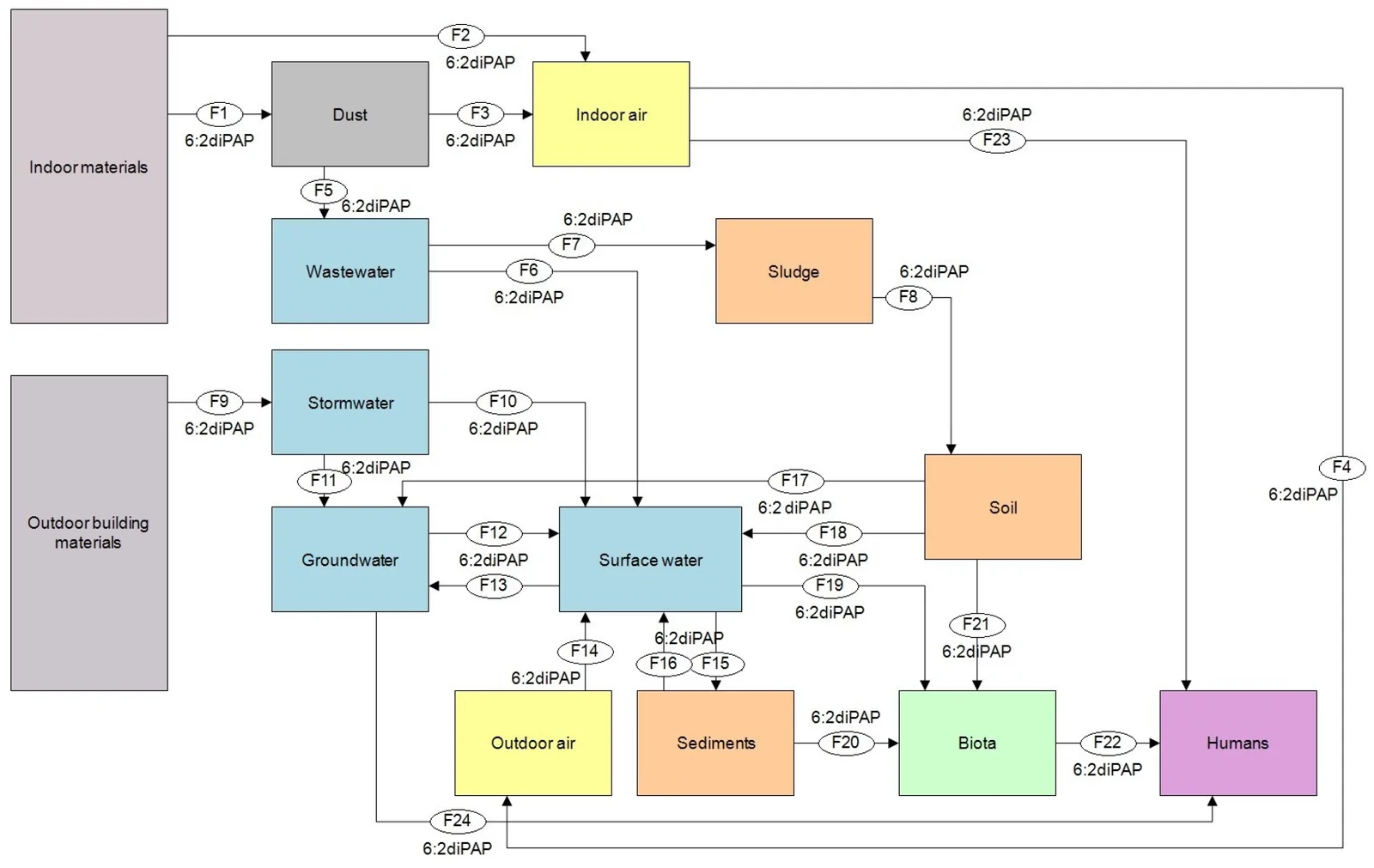
A conceptual model of 6:2 diPAP flows. F—flows. Transformation processes are not shown.

Figure 4 presents the conceptual model of 6:2 di PAP flows.

6:2 diPAP is released to the environment throughout the service life of construction products in which it is used as an additive rather than being chemically bound to the material matrix [39]. The model differentiates between indoor and outdoor construction applications as they have different patterns for substance release. Indoors, volatilization and the abrasion (F1–F3) of treated materials contribute to the presence of 6:2 diPAP in indoor dust and air, making these compartments important for human exposure [29,42,74,75] (see Table 2). Indoor dust may subsequently enter wastewater systems during cleaning activities (F5), while air exchange through ventilation transports airborne 6:2 diPAP from indoor spaces to the outdoor atmosphere (F4), potentially facilitating its wider redistribution (F14).

Wastewater treatment plants are not really designed to treat complex persistent organic compounds, and therefore allow for the distribution of 6:2 diPAP or its transformation products further into receiving water bodies (F6) or into the sewage sludge (F7) [76]. Biosolid application transfers 6:2 diPAP to agricultural soils (F8), where concentrations of 23.7–82.5 ng/g d.w. have been measured in sludge-amended soils [77].

Outdoor applications constitute an additional source through rain-induced wash-off from treated surfaces (F9) [39] followed by transport via stormwater runoff into receiving surface waters (F10) [22]. At the same time, infiltration and percolation processes may transfer 6:2 diPAP and its transformation products from soil to groundwater (F11–F13). Lysimeter experiments demonstrated that 23.2% of the initially applied 6:2 diPAP mass was transformed into PFCAs and recovered in leachates, whereas 36–45% of the parent compound remained in soil after two years [37]. The occurrence of 6:2 diPAP in tap water (0.24–1.52 ppt) and bottled water (0.2–3.8 ppt) indicates that groundwater contamination and subsequent human exposure through drinking-water abstraction (F24) are possible [78].

Once released into aquatic environment, 6:2 diPAP may remain dissolved in the water column, adsorb to suspended particles, or accumulate in bottom sediments (F15), which act as environmental sinks. However, sediments should not be regarded as a permanent endpoint because microbial activity and changing environmental conditions may promote remobilization or transformation of the compound (F16). Since 6:2 diPAP is a PFAS precursor, it can gradually transform into PFCAs, thereby extending its environmental persistence and increasing the potential for transport through aquatic systems [37,77]. Consistent with this transformation, ref. [79] observed that 6:2 diPAP and 8:2 diPAP concentrations in sediments were three to four orders of magnitude lower than those of terminal PFCAs and perfluoroalkanesulfonates (PFSAs).

Waters may also facilitate contaminant transfer to aquatic organisms (F19, F20), as evidenced by the detection of 6:2 diPAP in benthic worms at concentrations of 18–103 pg/g w.w. [46]. Contaminated soils represent an exposure pathway for terrestrial biota and vegetation (F21) [45]. Ultimately, these environmental pathways may contribute to human exposure (F22, F23), as demonstrated by the occurrence of 6:2 diPAP in human blood at concentrations of up to 0.056 ng/mL [80].

## 4. Discussion

The combination of the performed SFA and empirical screening under the Interreg project NonHazCity3 provides a dual perspective on the issue of hazardous chemicals in the environment due to their usage in building materials. While SFA demonstrates technological stocks and potential pathways of the selected substances, the field data generated by NonHazCity3 [22] serve as a reality check, confirming that these pathways are active and continuously contribute to the presence of the substances to the indoor and outdoor environments.

### 4.1. TCPP Technospheric Stock Accumulation, Environmental Exposure and Toxicological Relevance

The SFA for TCPP shows that the European system is dominated by technospheric accumulation, with nearly 70,804 t/year entering the anthropogenic stock, primarily within rigid PUR insulation boards, followed by flexible PUR foams and spray/1K foams. The large technospheric stock should not, however, be interpreted as environmentally insignificant. Even when the fraction released from individual products is relatively small, the large quantity of TCPP incorporated into long-lived building materials creates a substantial reservoir from which emissions can occur over an extended period.

The NonHazCity3 screening confirmed the environmental relevance of these pathways. TCPP was detected in all eight preschool dust samples in Västerås, with a median concentration of 0.665 μg/g [22]. Residential wastewater in Stockholm contained 5.5 μg/L TCPP, while wastewater treatment plant inlet water contained 3.6 μg/L, pointing to the fact that residential areas are the important source of TCPP. TCPP was also detected in 37% of analyzed stormwater samples, at concentrations up to 0.52 μg/L [22]. These observations provide empirical evidence that TCPP contained in building-related and consumer materials is not confined to the technosphere but contributes to contamination of indoor and outdoor environments.

The preschool dust results are particularly relevant from a human-exposure perspective. The median TCPP concentration reported in the NonHazCity3 screening campaign (0.665 µg/g) was lower than the median concentration of 1.66 µg/g reported by Giovanoulis et al. [29] in dust from 20 Swedish preschools sampled in 2018, corresponding to approximately 40% of the previously reported median. Nevertheless, TCPP was detected in all eight preschools investigated in Västerås, consistent with the 100% detection frequency reported by Giovanoulis et al. [29]. The latter study also reported a 95th percentile concentration of 39.4 µg/g, demonstrating considerable variability in TCPP contamination among preschool environments. Thus, although the concentrations observed in the NonHazCity3 screening were not exceptionally high compared with previous Swedish measurements, the consistent detection indicates that exposure to TCPP through indoor dust is widespread rather than restricted to highly contaminated locations.

This interpretation is supported by recent reviews of organophosphorus flame retardants (OPFRs) in indoor environments, which identify TCIPP/TCPP among commonly detected OPFRs and highlight indoor dust as an important medium for human exposure through ingestion, dermal contact and inhalation [87]. Giovanoulis et al. [29] further assessed children’s exposure to the chemicals detected in preschool dust through dust ingestion and reported hazard quotients below one for the substances considered, including after product-substitution measures. This finding indicates that the measured presence of TCPP should not by itself be interpreted as evidence of adverse health effects. Rather, it shows that this is a relevant exposure pathway that should be considered together with the substance’s toxicity and how often people are exposed to it.

From a toxicological perspective, this distinction between environmental occurrence and risk is important. Experimental and regulatory evidence indicates that TCPP can cause adverse effects across different biological levels. Early assessments, including the EU Risk Assessment Report, identified low acute toxicity but reported irritation potential and effects on target organs following repeated exposure [23]. The same assessment identified effects on fertility and development in experimental animals, while subsequent regulatory screening indicated the need for further consideration of TCPP in articles [23,24]. More recently, the US NTP Technical Report 602 reported some evidence of carcinogenic activity in male and female rats and male mice, and clear evidence of carcinogenic activity in female mice following chronic exposure to an isomeric mixture of tris(chloropropyl) phosphate [25]. Increased incidences of non-neoplastic lesions were also observed in the liver of male and female rats and female mice, as well as in the kidney of male mice [25]. Antonopoulou et al. reported cytotoxic and genotoxic responses in cultured human lymphocytes at the higher concentrations tested and observed adverse effects on several freshwater and marine microalgae species as well as bacteria [87]. These findings do not demonstrate that the environmental concentrations measured in the NonHazCity3 study cause comparable effects, because the experimental exposure conditions and concentrations differ substantially. Nevertheless, they demonstrate that TCPP has biological activity relevant to both human and environmental health.

The toxicological significance of TCPP emissions is better understood in terms of continuous exposure potential from widespread use and release rather than assuming acute toxicity from the measured environmental concentrations. Smaller, unsealed applications, such as flexible foams and 1K foams, may be disproportionately important for real-world exposure despite contributing less to the total technospheric stock. Physical abrasion, cutting, weathering and wear can increase the release of additive TCPP, while indoor dust provides a direct pathway for human exposure through ingestion, inhalation and dermal contact. The detection of TCPP in human matrices reported in various studies also demonstrates that exposure occurs in the general population [26,31,32].

The combination of the large anthropogenic stock, continuous release, widespread occurrence and experimentally observed hazardous properties supports the identification of TCPP as a substance of toxicological relevance in the built environment.

### 4.2. Leaching of Diuron from Facades and Aquatic Ecotoxicity Risks

The conceptual model identifies building facades as an important source of diuron to urban aquatic environments, with rainfall-driven wash-off representing the dominant release mechanism. The NonHazCity3 stormwater screening supports this pathway being one of the probable sources of diuron in waters: diuron was detected in 8% of samples from Stockholm and 36% from Turku, with the highest concentrations associated with areas dominated by relatively new wooden residential buildings. In one such residential area, diuron was detected in all samples and reached 1.9 μg/L, whereas it was not detected in the building-free control area. The measured peak concentration slightly exceeds the short-term threshold of 1.8 μg/L under Dir. 2013/39/EU [35], and exceeds the revised acute limit by seven times (MAC-EQS = 0.27 μg/L for inland surface waters) set in Dir. (EU) 2026/805 [88]. Also, the environmental concentrations compiled in Table 1 demonstrate that diuron released from building materials can occur at concentrations relevant to aquatic exposure. Particularly notable are concentrations measured in runoff from freshly treated facades, which reached the mg/L range. These concentrations are substantially higher than those typically observed in municipal wastewater and demonstrate the importance of building surfaces as localized sources of diuron to urban aquatic systems. The toxicological relevance is particularly pronounced for primary aquatic producers because diuron inhibits photosystem II and can impair photosynthetic activity in algae and aquatic macrophytes [38,42]. The measured high concentrations indicate that runoff from building areas can represent a potential acute ecological exposure scenario. However, these concentrations should not be interpreted directly as concentrations causing effects in receiving waters, because dilution, degradation and other environmental attenuation processes occur after release.

Still, diuron’s persistence makes it even more environmentally relevant. The substance can remain in soils and sediments for prolonged periods, while degradation is substantially slower under dark aquatic conditions than under sunlight [65,70]. Its transformation product 3,4-DCA may also be of concern because of its greater ecotoxicity than the parent compound [32,33]. Thus, the toxicological significance of facade emissions is not restricted to the initial concentration pulse following rainfall but may include repeated exposure and transformation processes within receiving environments.

In contrast to TCPP and 6:2 diPAP, the evidence available for diuron supports a comparatively direct link between a building-related source, measured environmental occurrence and ecological hazard. The results therefore provide an example of how the choice of construction material or surface treatment can create a chemical exposure in aquatic ecosystems.

### 4.3. 6:2 diPAP Release, Precursor Transformation and Exposure Pathways

The NonHazCity3 field data support both indoor and outdoor pathways of 6:2 diPAP to the environment. 6:2 diPAP was among the most abundant PFAS detected in dust samples from preschools in Stockholm (the median value 253.33 ng/g) and Västerås (up to 87.02 ng/g), demonstrating that indoor materials can represent an exposure source [22]. In case of Västerås, it was assumed to be related to floor material. Also, the PFAS might have been added unintentionally during cleaning and floor care: the old floor polish was removed in a few sampling places some years ago and a new base primer and wax, which are the probable source of PFAS, were added on the floor surface.

In the outdoor compartment, PFCAs, which represent primary and secondary degradation products of 6:2 fluorotelomer procursors like 6:2 diPAP, were detected at varying concentrations in stormwater across all screened municipalities (Stockholm, Helsinki, Turku, and Västerås). PFHxA was detected in 73–100% of samples (up to18 ng/L), PFHpA in 73–100% (up to 11 ng/L), and PFPeA in 57–100% (up to 19 ng/L) [22].

Because PFAS are used in very small quantities, they bypass regulatory thresholds for disclosure, such as under REACH [12] or building databases like BVB [18], remaining invisible in supply chains but present in environmental runoff.

The toxicological relevance of these findings arises from the combination of exposure potential and persistence. 6:2 diPAP is a PFAS precursor capable of transformation into persistent PFCAs. In the present conceptual model, this means that the environmental burden associated with a building material cannot necessarily be represented by measurements of the parent compound alone. Transformation may shift the chemical composition of the contamination while maintaining or extending its persistence and environmental mobility.

The literature data compiled in Table 2 demonstrate the occurrence of 6:2 diPAP in indoor dust, drinking-water matrices and human blood, although these observations do not establish that buildings are the direct source of the measured human body burdens. The broader PFAS literature indicates that persistent and mobile PFAS can move between indoor and outdoor environments, groundwater, surface waters, biota and human populations through multiple interconnected pathways [89]. Therefore, building-related emissions should be considered one potential contributor within a larger and interconnected PFAS exposure system.

NonHazCity3 analysis should not be interpreted as demonstrating that the measured concentrations of 6:2 diPAP cause adverse health effects. The significance of the findings is instead that construction and interior materials can introduce PFAS precursors into exposure pathways from which persistent transformation products may subsequently arise. This supports a precautionary approach in which precursor emissions are considered alongside the toxicity and persistence of their potential transformation products.

### 4.4. Toxicological Significance and Implications for Chemicals Management

A common feature of the three case studies is that environmental relevance cannot be determined from production or use quantities alone. The substances differ markedly in their hazard profiles and environmental behavior, and consequently the toxicological significance of their emissions arises through different mechanisms. Taken together, the cases illustrate the importance of considering hazard, exposure and environmental fate jointly when evaluating chemicals used in construction products. This study does not provide quantitative human-health or ecological risk quotients; nevertheless, the combination of substance-flow modeling and NonHazCity3 measured environmental occurrence demonstrates that the identified emission pathways are active and can connect building materials with human and environmental exposure.

The cases also highlight the problem of regrettable substitution. TCPP was introduced to replace brominated flame retardants and then TCEP [22,26], but it turns out that TCPP also causes concerns with regard to its own hazards, emissions to, and presence in the environment. Diuron continues to be evaluated under BPR despite its non-renewal as an active substance for plant protection products [13,34]. 6:2 diPAP and other short-chain telomers were introduced as “safer” alternatives to long-chain PFA, but they are precursors of PFCAs and thus persist in the environment [90]. A substitution strategy based solely on replacing a restricted substance with another substance performing the same technical function may shift rather than eliminate environmental and toxicological concerns. From a risk-prevention perspective, upstream source control is consequently preferable to relying exclusively on downstream treatment. Material selection that avoids hazardous additive flame retardants, minimizes biocide-treated building surfaces and prevents the use of persistent fluorinated additives can reduce emissions at their source. Improved chemical transparency and digital building-material logbooks would further enable identification of hazardous substances during renovation, demolition and recycling. Such measures are particularly important for long-lived buildings because chemicals incorporated into construction products may remain in the built environment for decades and can subsequently enter environmental or recycling pathways.

### 4.5. Study Limitations

While this study provides a screening-level evaluation connecting chemical flows from buildings to environmental exposure, several limitations must be acknowledged.

SFA and conceptual models rely on specific model assumptions and are subject to data uncertainty. For the TCPP SFA, pre-existing technospheric stocks were excluded to manage uncertainty; therefore, the analysis solely reflects annual inputs and transient flows rather than total historical accumulation. For diuron and 6:2 diPAP, a lack of comprehensive production volumes and emission factors restricted the analysis to conceptual models rather than full quantitative mass balances.

When interpreting the environmental relevance of these flows, a clear distinction must be made between source water and receiving water. Building runoff or local stormwater represent the initial emission pulse. These source concentrations will undergo substantial dilution, sorption to sediments, and biotic or abiotic degradation before and within receiving water bodies. Therefore, source measurements indicate potential loading and acute localized hazards, but do not represent the final exposure concentrations for wider aquatic communities.

Also, the study lacks a full quantitative risk assessment. Although the analysis combines empirical occurrence data with toxicological characteristics to identify relevant exposure pathways, it does not calculate specific human health or ecological risk quotients. The findings should be interpreted as a hazard-based screening that highlights toxicologically relevant emission pathways rather than a definitive quantification of absolute risk to specific populations or ecosystems.

## 5. Conclusions

The combination of SFA and environmental screening by NonHazCity3 demonstrated that buildings should be considered not only reservoirs of substances of concern but also sources contributing to environmental exposure and potential toxicological pressure. The three case studies showed that, in addition to the quantities used, the environmental significance also depends on how substances are released, transported, transformed, and finally become available for exposure to organisms and humans.

For TCPP, the quantitative SFA showed that the European system is dominated by accumulation in the technosphere, with approximately 70,804 t/year incorporated into products, mainly rigid polyurethane insulation materials. Despite the relatively small release fractions, the model estimated net annual accumulation of 444 tonnes in aquatic systems and 64 tonnes in soils. The detection of TCPP in indoor dust, wastewater, and stormwater confirms that this large technospheric stock is accompanied by continuous environmental releases. Together with its documented chronic toxicity and carcinogenic potential, these findings identify TCPP as a substance of toxicological relevance in the built environment.

For diuron and 6:2 diPAP, the conceptual models, supported by NonHazCity3 screening data and literature evidence, identified different but environmentally relevant pathways. Diuron is released mainly through rainfall-driven wash-off from treated facades and can result in repeated exposure of aquatic primary producers. The detection of diuron in stormwater, including at concentrations exceeding relevant environmental quality thresholds in a comparatively new district of wooden construction, supports the importance of building surfaces as potential emission sources and highlights a potential ecotoxicological concern. For 6:2 diPAP, both indoor and outdoor pathways were identified, with occurrence in indoor dust and mainly its transformation products (PFCAs) occurring in stormwater. The formation of persistent PFCAs is particularly relevant because it can extend the environmental persistence and mobility of fluorinated contamination and thereby prolong potential human and environmental exposure.

A meaningful assessment therefore requires consideration of the complete pathway from material use to environmental occurrence, transformation, and exposure, together with the hazard characteristics of the substances involved. The combination of SFA, environmental monitoring, and toxicological information provides a useful screening approach for identifying substances and applications that call for closer attention. As buildings have a long service life, priority should be given to preventing hazardous substances from entering construction products in the first place, while improving chemical transparency to support safer renovation, demolition, and recycling.

## Figures and Tables

**Table 1:** Reported diuron concentrations in building runoff and environmental matrices, with environmental relevance and exposure implications.

Environmental Compartment/Matrice	Location	Diuron Concentration	Reference	Exposure Implications
**Source and building runoff**				Potential acute exposure of aquatic primary producers. Indicates the highest release initially, and then continued release from treated facades and recurrent aquatic exposure
Model house facade runoff:	Switzerland	Up to 39.5 mg/L	[5]	
initial burst phase				
later consistent level within one year	Switzerland	1–5 mg/L		
Facade runoff, plastered and painted facades	Germany	Mean 900 µg/L	[3]	
Roof runoff, small painted roof components	Germany	<0.5 µg/L	[3]	
Eluate from a recently repainted inner roof facade	Germany	2.7 µg/L	[69]	
Eluate from an older northern roof facade	Germany	48 ng/L	[69]	
**Urban stormwater and infiltration systems**				Potentially ecologically relevant exposure; concentrations may exceed the EQS
Storm-sewer runoff from residential sites, Berlin	Germany	0.2–2.5 µg/L	[3]	
Urban stormwater	Values compiled from multiple studies, Europe	Up to 12 µg/L	[8]	
Standing water in an infiltration swale, Freiburg		174 ng/L	[69]	Potential retention in infiltration systems and transfer to groundwater
Urban stormwater	BSR cities (Sweden, Finland)	Up to 1.9 µg/L	[22]	
**Wastewater and sludge**				Occurrence in municipal wastewater. Incomplete removal and continued discharge to receiving waters
Municipal wastewater-treatment-plant influent during dry weather	Denmark and Sweden	4–39 ng/L	[70]	
Municipal WWTP influent, two treatment plants	Germany	23–68 ng/L	[43]	
Municipal WWTP effluent, two treatment plants	Germany	25–182 ng/L	[43]	
Municipal WWTP influent, separate study	France	31 ng/L	[59]	
Sludge	Germany	24 ng/g TSS	[43]	Potential terrestrial exposure if sludge is applied to agricultural soils
**Surface and marine water**				Potential exposure of aquatic communities
Rivers and other surface waters, literature compilation	Values compiled from multiple studies, Europe	Up to 8.6 µg/L	[8]	
Aquatic environments	Japan	3.05 µg/L	[66]	
Coastal and marina waters	Netherlands	>430 ng/L	[71]	
**Groundwater**				Potential long-term contamination of groundwater resources
Groundwater	United Kingdom	Up to 140 ng/L	[65]	
Upgradient of the infiltration system	Germany	Mean 1.1 ng/L	[72]	
Downgradient of the infiltration system	Germany	Mean 8.8 ng/L	[72]	Indicates increased concentration downstream of infiltration
**Soil and sediment**				
Soil of stormwater infiltration systems	Germany and France	168 µg/kg	[73]	Potential terrestrial exposure

**Table 2:** Occurrence of 6:2 diPAP in in environmental and human matrices reported in the literature and implications for exposure.

Environmental Compartment/Matrice	Location	Concentration	Reference	Exposure Implications
**Indoor dust**				Potential ingestion/inhalation exposure, particularly relevant for indoor environments
Preschool dust	Sweden	1140 ng/g	[29]	
Children’s bedrooms dust	Finland	675 ng/g (average)	[74]	
Residential indoor dust	Norway	742 ng/g (average)	[75]	
Residential indoor dust profiles	United States and Canada	113 ng/g (median)	[41]	
Fire stations indoor dust profiles	United States and Canada	287 ng/g (median)	[41]	
Residential indoor dust	China	0.32–69.7 ng/g	[42]	
Hotel indoor dust	China	3.15–183 ng/g	[42]	
Hotel indoor dust	China	1.08–67.4 ng/g	[81]	
Preschool dust	BSR cities (Sweden)	629.93 ng/g	[22]	
**Indoor air**				Potential inhalation exposure
Cinema indoor air	China	125 pg/m^3^ (dominant diPAP)	[42]	
Offices and auto salons	China	3.20–15.8 pg/m^3^ (dominant diPAP))	[42]	
Hotel indoor air	China	<1–6.7 pg/m^3^	[81]	
**Water**				
Surface water	China	Up to 0.134 ng/L	[79]	Aquatic exposure and precursor transport
Tap water	Australia	Average 0.60 ppt, range 0.24–1.52 ppt	[78]	Potential drinking-water exposure
Bottled water	Australia	Average 1.4 ppt, range 0.2–3.8 ppt	[78]	
**Sewage sludge**				Transfer to soil through sludge application
Municipal sewage sludge	Sweden	2.0 ng/g	[82]	
Municipal sewage sludge	France	1.7 ng/g	[83]	
Municipal sewage sludge	Sweden	5.6 ng/g	[83]	
Municipal sewage sludge	United States	27.2 to 750 ng/g	[83]	
Municipal sewage sludge	Canada	0.5 μg diPAP/g sludge	[36]	
Municipal sewage sludge	China	11.6 ng/g d.w.	[46]	
**Soil**				Potential terrestrial exposure and plant uptake
Sewage sludge-amended soil	Canada	23.7 ng/g dw	[77]	
Topsoil from lysimeter study after 2 years	Germany	730 μg/kg DM	[37]	
**Landfill**				Potential release from waste-management systems
Municipal landfill leachate	United States	5.7–49 ng/L	[44]	
**Living organisms**				
Humans (blood serum)	Norway	0.018–0.14 ng/mL	[84]	
Biota (worm)	China	18–103 pg/g w.w.	[46]	Evidence of uptake by aquatic biota
Humans (blood)	China	<0.0016–0.013 ng/g	[46]	Evidence of human exposure; source attribution not established
Human serum	United States	1.9 μg/L	[85]	
Food (fish, meat, eggs, and pasta)	Sweden	0.2 and 428 pg/g	[86]	

## Data Availability

The original contributions presented in this study are included in the article. Further inquiries can be directed to the corresponding author.

## Supplementary Materials

The following supporting information can be downloaded at: https://www.mdpi.com/article/10.3390/toxics14090811/s1, Table S1: Explanation of flows. References [23,50,51,57,58] are cited in the Supplementary Materials.

## Abbreviations

The following abbreviations are used in this manuscript:

1K	One-component
3,4-DCA	3,4-dichloroaniline
6:2 diPAP	Bis(2-(perfluorohexyl)ethyl) phosphate
BPR	Biocidal Products Regulation
CPR	Construction Products Regulation
ECHA	European Chemicals Agency
EQS	Environmental Quality Standard
F	Flow
NonHazCity3	Project acronym: “Reducing hazardous substances in construction to safeguard the aquatic environment, protect human health and achieve more sustainable buildings”
OPEs	Organophosphate esters
OPFRs	Organophosphorus flame retardants
PFAS	Per- and polyfluoroalkyl substances
PFCAs	Perfluoroalkyl carboxylic acids
PFHpA	Perfluoroheptanoic acid
PFHxA	Perfluorohexanoic acid
PFPeA	Perfluoropentanoic acid
PFOA	Perfluorooctanoic acid
PUR	Polyurethane
REACH	The Regulation on the registration, evaluation, authorisation and restriction of chemicals
SFA	Substance flow analysis
TCEP	Tris(2-chloroethyl) phosphate
TCPP	Tris(2-chloro-1-methylethyl) phosphate
