# Supplementary material for: From Buildings to the Environment: Flows, Exposure Pathways, and Toxicological Relevance of Tris(2-Chloro-1-Methylethyl) Phosphate (TCPP), Diuron, and Bis(2-(Perfluorohexyl)Ethyl) Phosphate (6:2 diPAP)
Source: Toxics. 2026 Sep 11;14(9):811. doi: 10.3390/toxics14090811 (PMC13611191; doi:10.3390/toxics14090811)
Supplement: Supplementary file 1 [file toxics-14-00811-s001.zip › toxics-4480956-supplementary.pdf]

SUPPLEMENTARY MATERIAL

Table S1. Explanation of flows: assumptions made, references, entered and reconciled values, including uncertainties (coefficients of variation (CV)).

**Flow**	**Description**	**Assumptions and calculations**	**Refe-rences**	**Reconciled values**		**Values entered in STAN**	
				**Amount, TCPP t/year**	**CV, %**	**Estimated amount, TCPP t/year**	**CV, %**
F1: Imported	TCPP imported to European Union	1) According to [1] 1064, import of tris(1-chloro-2-propyl) phosphate (TCPP) and triethyl phosphate (TEP) during the investigation period (1 July 2022 to 30 June 2023) amounted to 56,000–66,000 tonnes. As TEP constituted less than 5 % of the EU consumption [2], 95% was assigned to TCPP. Thus, import of TCPP was within 53,200–62,700 t/year, or 57,950 t/year as an average of this.<br>2) Another way of calculations: Total Union consumption of TCPP and TEP was 71,000-81,000 t/year [1] ⇨ 76,000 t/year as average. Therefore, TCPP consumption (95%) was 72,200 t/year. Imports, mainly from China, constituted 80% of market share [2]: 72,200 x 80% = 57,760 t/year.<br>3) For cross checking: In our model: F1+F3-F6-F7 = 72,304 t/year (vs ~ 72,200 t/year [1]).	[1,2]	57,030.25*	8.4	57,950	15
F2: Produced	TCPP produced in European Union	The total production during the investigation period was established at around 18,274 tonnes.	[1]	18,228.29	10.4	18,274	15
F3: Produced	A produced flow to consumption	A produced flow to consumption, considering release from production and compounding/system formulation	[1]	18,219.15	10.5	18,264.86	15

F4: Release	Release from TCPP production and compounding/ system formulation to the air	Losses to the air 0.025%.	[3]	4.57	30.6	4.57	30
F5: Release	Release from TCPP production and compounding/ system formulation to the water	Losses to the wastewater 0.025%.<br>Even if TCPP enters conventional treatment processes, they do not effectively remove or treat it. Therefore ,TCPP flows are directly diverted to aquatic compartment.	[3]	4.57	30.6	4.57	30
F6: Export	Export of TCPP from EU	The volume of exports of the EU producers.	[1]	2333.49	12.9	2332	15
F7: Confidential/ unknown	The share of usage, which could not be allocated to defined use sectors	Information on the use of < 2.5% TCPP was confidential or unknown; this fraction could not be allocated to defined use sectors and is therefore shown as leaving the system boundary. Considering known uses and total consumption volume, confidential/ unknown was calculated: 72,200 t/year x 2.2%.	[3]	1589.69	15.1	1588	15
F8: For rigid foam	TCPP used to produce rigid foam	66.5%, or two thirds, from the total TCPP consumption.	[1,3]	48,688.77	9.1	48,013	15
F9: Release	Release to the air from rigid foam production	Release rate 4.8E-06%.	[3]	0.0023	30.4	0.0023	30
F10: Release	Release to the water from rigid foam production at larger sites	Release rate 4.8E-06%. Larger sites account for 30% of the tonnage of TCPP, added directly by the foamers.	[3]	0.00069	30.4	0.00069	30
F11: Release,	Release to the	Release rate from small sites to water 0.0100048%. 70% of	[3]	3.363	29.7	3.363	30

from small sites	water from rigid foam production at small sites	TCPP is added via systems; small producers tend to use systems.					
F12: In rigid PUR insulation boards	TCPP in rigid PUR insulation boards	Equals to TCPP for rigid foam production minus releases at production.	–	47,965.23	9.3	48,099.63	15
F13: Weathering & wear	Weathering & wear from rigid PUR insulation boards	Release rate 0.8%, loss to soil from weathering and wear in service (cutting at construction sites) or at disposal.	[3]	384.08	31.2	384.08	30
F14: In rigid foam scrap	Scrap of rigid foam production that is recycled by adhesive pressing	1.5% of the rigid foam tonnage is recycled by adhesive pressing.	[3]	720.18	21.2	720.20	30
F15: Release	Release to the air from adhesive pressing	Release rate 0.1%.	[3]	0.7202	30.5	0.7202	30
F16: Release	Release to the water from adhesive pressing	Release rate 0.1%.	[3]	0.7202	30.5	0.7202	30
F17: In rigid panels	TCPP in rigid panels	Equals to TCPP in scrap that was used for adhesive pressing minus releases at processing.	–	718.73	21.2	718.75	30
F18: Release	Release to the air from rigid panels in service	Release rate 3.33E-03%.	[3]	0.0239	30.1	0.0239	30
F19: Release	Release to the water from rigid panels in service	Release rate 3.33E-03%.	[3]	0.0239	30.1	0.0239	30
F20: For flexible	TCPP used to	17% from the total TCPP consumption.	[3]	12,299.88	11.7	12,274	15

foam	produce flexible foam						
F21: Release	Release to the air, mainly from curing and storage, at flexible foam production sites	Release rate 1.2E-04%.	[3]	0.0147	29.9	0.0147	30
F22: Release	Release to the water, mainly from curing and storage, at larger flexible foam production sites	Release rate 1.2E-04%. Larger sites account for 85% (70% very large and 15% assumed as "large") of the tonnage of TCPP.	[3]	0.0125	30.4	0.0125	30
F23: Release, from small sites	Release to the water at small flexible foam production sites	Release rate 0,01012%. Small sites assumed to account for 15% of the tonnage of TCPP.	[3]	0.1863	30.1	0.1863	30
F24: To solid waste	Loss of foam block sides to solid waste	0.2% TCPP to solid waste.	[3]	24.55	30.1	24.55	30
F25: In flexible foam	TCPP in flexible PUR foam	Equals to TCPP for flexible foam minus releases.	–	12,275.11	11.8	12,249.24	15
F26: Release	Release to the air from cutting and using in manufacture of furniture	Release rate 0.0002%.	[3]	0.0245	29.8	0.0245	30
F27: Release	Release to the water from cutting	Release rate 0.0002%.	[3]	0.0245	29.8	0.0245	30

	and using in manufacture of furniture						
F28: In flexible foam in articles	TCPP in flexible foam in articles	Equals to TCPP in flexible foam minus releases and TCPP in scrap foam.	–	10,437.39	14	10,411	30
F29: Release	Release to the air from flexible foam in articles in service	Release rate 9.6E-03%.	[3]	0.9995	30	0.9995	30
F30: Weathering & wear	Weathering & wear from flexible PUR foam	Release rate 0.8%. It is associated with physical weathering and wear and hence release of TCPP to the air from foam particles.	[3]	83.29	30	83.29	30
F31: In scrap foam	TCPP in scrap foam for recycling	It is assumed that 10.5% of TCPP in furniture foam is used for rebonding, and 4.5% is recycled as loose crumb.	[3]	1837.68	18.1	1837.39	30
F32: Release	Release to the air from rebonding and recycling into loose crumb	Release rate 4E-04%.	[3]	0.0073	30.1	0.0073	30
F33: In rebonded foam	TCPP in rebonded foam	Based on assumption under F34, 70% of foam scrap is rebonded.	[3]	1286.41	24.8	1286.16	30
F34: Release	Release to the air from rebonded foam during service life	Release rate 9.6E-03%.	[3]	0.1235	30.0	0.1235	30
F35: Weathering & wear	Weathering & wear from rebonded foam, release to the air	Release rate 0.8%.	[3]	10.29	30.0	10.29	30

F36: In loose crumb	TCPP in loose crumb	Based on assumption under F34, 30% of foam scrap is recycled as loose crumb.	[3]	551.26	29.1	551.21	30
F37: Release	Release to the air from loose crumb during service life	Release rate 0.096%.	[3]	0.5292	30.2	0.5291	30
F38: Weathering & wear	Weathering & wear from loose crumb, release to the air	Release rate 0.8%.	[3]	4.410	29.5	4.410	30
F39: For spray & 1K foams	TCPP used for spray foams and 1K foams	14.3% (9.6% spray foams and 4.7% 1K foams) from the total TCPP consumption.	[3]	10,338.58	10.5	10,320	15
F40: Release	Release to the air from foaming in situ	Release rate 0.096%.	[3]	9.910	30.0	9.911	30
F41: In foam	TCPP in foam	Equals to TCPP in spray foams and 1K foams minus release during foaming in situ	–	10,328.67	10.5	10,314	15
F42: Deposition over land	TCPP deposition from the air over land	Transfer between environmental compartments for TCPP is consistent with the EU risk assessment for TCPP as well as other studies, which, based on fugacity modelling Level III, conclude that atmospheric TCPP predominantly deposits to soil, that there is relatively little movement between soil and water, and that adsorption to sediment from the water column is very low. We assumed that 90.5% of atmospheric TCPP deposits to land (F42), 9.5% directly to surface waters (F43), 9.1% of soil-bound TCPP is transferred to water via leaching/runoff per year (F44), and 0.32% of dissolved/ particle-associated TCPP in the water column is transferred to sediment per year (F45).	[3-5]	104.00	60.0	104.00	60
F43: Deposition	TCPP deposition from the air to the waters			10.92	60.0	10.92	60
F44: Leaching/ runoff	TCPP from the soil into the waters			44.42	60.0	44.42	60
F45: Particle settling	TCPP from the water column into the sediments			0.2055	60.0	0,2055	60

*- Two decimal places were retained for all flows in Figure 1 of the manuscript to make small changes in the mass balance visible, as the contribution of some flows would not be apparent if the values were rounded to whole numbers. Consequently, the largest values in Table S1 are also displayed with two decimal places, not because such a high level of precision is required, but to maintain a consistent number format and facilitate matching the values presented in Figure 1 with those reported in the Supplementary material Table S1. Some of the smallest flows still appear as zero in Figure 1 due to rounding to two decimal places; however, their precise values are provided in this Supplementary Table.

This trade-off results from the number-formatting options available in the STAN software. The software allows either a fixed number of decimal places or a format based on significant figures. The latter results in greater rounding of the largest values, which can obscure small changes in the mass balance because the smaller flows have only a minor effect on the larger flows. Therefore, the fixed two-decimal-place format was selected to provide a consistent representation of the flows and to make small mass-balance changes more readily identifiable.

References

1. European Commission. *Commission Implementing Regulation (EU) 2024/1064 of 9 April 2024 Imposing a Provisional Anti-Dumping Duty on Imports of Certain Alkyl Phosphate Esters Originating in the People's Republic of China*; European Union: Brussels, Belgium, 2024. Available online: https://eur-lex.europa.eu/legal-content/EN/ALL/?uri=CELEX%3A32024R1064 (accessed on 20 December 2025).
2. European Commission. *Commission Implementing Regulation (EU) 2024/2415 of 12 September 2024 Imposing a Definitive Anti-Dumping Duty and Definitively Collecting the Provisional Duty Imposed on Imports of Certain Alkyl Phosphate Esters Originating in the People's Republic of China*; European Union: Brussels, Belgium, 2024. Available online: https://eur-lex.europa.eu/eli/reg_impl/2024/2415/oj/eng (accessed on 20 December 2025)..
3. European Commission. *European Union Risk Assessment Report: Tris(2-Chloro-1-Methylethyl) Phosphate (TCPP)*; European Communities: Brussels, Belgium, 2008. Available online: https://echa.europa.eu/documents/10162/17228/trd_rar_ireland_tccp_en.pdf (accessed on 20 December 2025).
4. Government of Canada. Draft Screening Assessment Certain Organic Flame Retardants Substance Grouping. Available online: https://www.canada.ca/en/environment-climate-change/services/evaluating-existing-substances/draft-screening-assessment3.html (accessed on 1 September 2026).
5. EPA. *TSCA Work Plan Chemical Problem Formulation and Initial Assessment*; Environmental Protection Agency: Washington, DC, USA, 2015. Available online: https://www.epa.gov/assessing-and-managing-chemicals-under-tsca/tsca-work-plan-chemical-problem-formulation-and-0 (accessed on 1 September 2026).
